# Explosive transitions to synchronization in networks of frequency dipoles

**DOI:** 10.1371/journal.pone.0274807

**Published:** 2022-09-20

**Authors:** Liuhua Zhu, Shu Zhu

**Affiliations:** 1 School of Physics and Telecommunication Engineering, Yulin Normal University, Yulin, Guangxi, China; 2 Guangxi Colleges and Universities Key Laboratory of Complex System Optimization and Big Data Processing, Yulin Normal University, Yulin, Guangxi, China; 3 Optoelectronic Information Research Center, Yulin Normal University, Yulin, Guangxi, China; Rey Juan Carlos University, SPAIN

## Abstract

We reveal that an introduction of frequency-weighted inter-layer coupling term in networks of frequency dipoles can induce explosive synchronization transitions. The reason for explosive synchronization is that the oscillators with synchronization superiority are moderately suppressed. The findings show that a super-linear correlation induces explosive synchronization in networks of frequency dipoles, while a linear or sub-linear correlation excites chimera-like states. Clearly, the synchronization transition mode of networks of frequency dipoles is controlled by the power-law exponent. In addition, by means of the mean-field approximation, we obtain the critical values of the coupling strength within and between layers in two limit cases. The results of theoretical analysis are in good agreement with those of numerical simulation. Compared with the previous models, the model proposed in this paper retains the topological structure of network and the intrinsic properties of oscillators, so it is easy to realize pinning control.

## Introduction

Synchronization is widely distributed in human society and natural environment. Synchronization in human society requires a unified command driven by external factors. For instance, when the monitor suddenly calls “stand up” in class, everyone stands up in unison, which is the synchronization mechanism under a unified command. However, the formation of synchronization in natural environment mainly depends on the cohesion of the system itself. In order to grasp the synchronous behaviors of complex systems, the synchronization dynamics of coupled phase oscillators has been widely studied in recent years [1–4]. Among the collective phenomena observed in these systems, the chimera states of coupled phase oscillators have aroused great interest of researchers [5–13]. The chimera state here refers to a transient state in the process of synchronization transition, in which some oscillators are locked and the remaining ones are in the drift state, that is, a coexistence state of synchronization and drifting.

In the past, researchers always thought that the synchronization in networks of coupled oscillators was a continuous and gradual phase transition process. This one-sided opinion was not ended until an explosive synchronization was found [14]. The so-called explosive synchronization means that the mode of synchronization transition is sudden without any omen. In the relation curve of order parameter versus coupling strength, the order parameter will jump sharply when the coupling strength increases to the critical value of the system. Meanwhile, the system transits abruptly from synchronization to incoherence state as the coupling strength is decreased, and the forward and backward continuations do not overlap, which results in a hysteresis loop [15–24].

In the field of electro-magnetism, how to analyze the polarization phenomenon of dielectric, the first thing is to discretize the dielectric into a system composed of multiple electric dipoles. In medical research, some dipole source models that produce scalp potential are established to speculate the source of electrical activity in the brain [25]. In industrial automation devices, when the ferromagnetic target is far enough from the sensor, it can be regarded as a magnetic dipole [26]. Inspired by these ideas, we try to discretize a complex system into a network composed of frequency dipoles. A double-layer network is considered because only one single-layer network is not enough to place multiple pairs of frequency dipoles. In this way, we can use the tool of complex network to study the dynamical behavior of complex systems [27].

Recently, the synchronization dynamics of multiplex networks has been deeply and carefully studied by employing different methodologies, for instance, phase-shift [28], adaptive inter-layer coupling [29], and inter-layer Hebbian plasticity [30]. As for the choice of coupled oscillators, the Kuramoto oscillator is the most mature and widely used tool in the research field of synchronization theory [31, 32], so it has also become our first choice. We use a double-layer network composed of two globally connected layers as the substrates, in which the upper and lower oscillators form frequency dipoles.

The follow-up parts of this paper are organized as follows. First of all, some basic notions on the frequency dipoles are introduced. In the next place, we give the main results of numerical simulations. Once again, the theoretical analysis and numerical simulation are carried out for the critical values of coupling strength within and between layers in two limit cases. Finally, we summarize our main findings and discuss open problems.

## Materials and methods

### Description of network model

We consider a two-layer network, in which each layer is composed of *N* globally connected nodes. In order to realize the connection between the two layers, *N* edges are added between the nodes with the same label in the upper and lower layers. After this step, a two-layer network similar to the pavilion structure is completed. The number of edges of the whole network is *N*^2^.

To explore the dynamics behavior of this network, each node in the network is embedded with a variant Kuramoto oscillator. The natural frequency of the *i*th oscillator in the upper layer is set to $\omega_{i}^{u}=-1+2\left(i-1\right)/\left(N-1\right)$. The value of $\omega_{i}^{l}$ in the lower layer is set to $\omega_{i}^{l}=-\omega_{i}^{u}$, so a pair of frequency dipoles are formed. Here *u* and *l* are identifiers of the upper and lower layers, respectively. The dynamics of the *i*th oscillator in the network is governed by the following equations,

$$\begin{array}{c} \frac{d\phi_{i}^{u}}{dt}=\omega_{i}^{u}+\frac{\mu }{N}\sum_{j=1}^{N}sin\left(\phi_{j}^{u}-\phi_{i}^{u}\right)+\lambda |\omega_{i}^{u}{|}^{\beta }sin\left(\phi_{i}^{l}-\phi_{i}^{u}\right),\\ \frac{d\phi_{i}^{l}}{dt}=\omega_{i}^{l}+\frac{\mu }{N}\sum_{j=1}^{N}sin\left(\phi_{j}^{l}-\phi_{i}^{l}\right)+\lambda |\omega_{i}^{l}{|}^{\beta }sin\left(\phi_{i}^{u}-\phi_{i}^{l}\right), \end{array}$$ (1)

where *i* = 1, 2, 3, …, *N*. The parameter *ϕ*_*i*_ is the instantaneous phase of the *i*th oscillator. The initial phase of each oscillator is randomly assigned within the range [−*π*, *π*]. The nonnegative parameter *μ* represents the intra-layer coupling strength, whereas the inter-layer coupling strength is denoted with λ.

Three types of positive correlations, i.e., *β* = 1, 0 < *β* < 1 and *β* > 1 are considered in this paper, which are also known as linear, sub-linear, and super-linear correlations, respectively. For better comparison, we supplement the case of *β* = 0, which corresponds to the homogeneous coupling.

In this paper, the size of each layer is set to *N* = 200 for numerical simulations, unless otherwise specified, because we find that the results described below do not change significantly for larger network sizes. The number of connected edges of each oscillator in these networks is constant, i.e., *k* = 200.

## Results

In this part, we reveal the detailed characteristics of frequency synchronization of coupled oscillators. The commonly used method is to introduce the effective frequency, which is defined as follows [14],

$$\begin{array}{c} \;\Omega_{i}^{u(l)}=\frac{1}{T}\int_{t_{0}}^{t_{0}+T}{\dot{\phi_{i}}}^{u(l)}\left(\tau \right)d\tau . \end{array}$$ (2)

where *t*_0_ is the relaxation time and *T* is the length of time used to obtain the average.

### Emergence of chimera-like state

**Fig 1:**
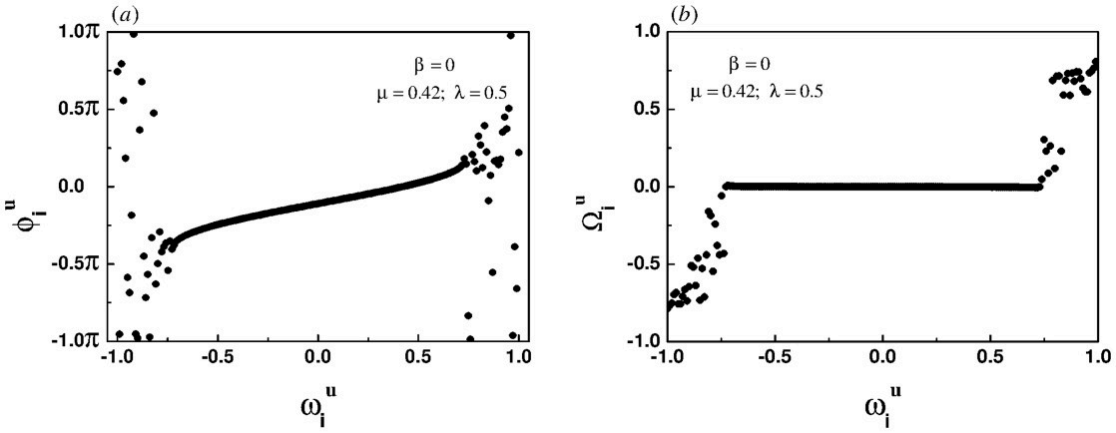
Snapshots of the instantaneous phases (left panel) and the effective frequencies (right panel) for the homogeneous coupling when the intra-layer and inter-layer coupling strength are set to 0.42 and 0.5, respectively.

Fig 1a and 1b show the snapshots of instantaneous phases and effective frequencies of all oscillators in the upper layer under the case of homogeneous coupling, respectively. The effective frequencies of the oscillators in the central region of the frequency spectrum tend to be the same, but the effective frequencies of the oscillators at both ends of the spectrum fluctuate greatly, which means that there is a chimera-like state. It is worth noting that the phenomenon is not a chimera state in the real sense. In fact, the chimera state is a unique synchronization phenomenon in a system with identical oscillators, whereas these oscillators in this paper are heterogeneous. Therefore, the phenomenon appearing in Fig 1b is called a chimera-like state.

**Fig 2:**
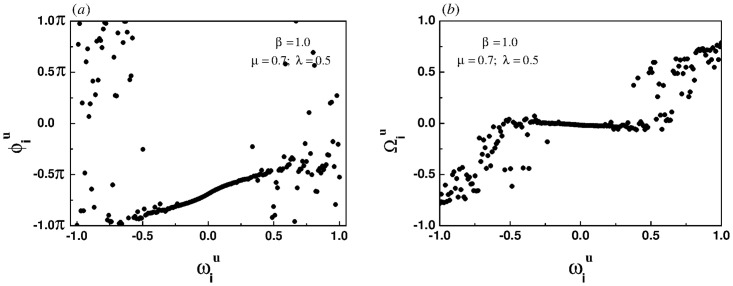
Snapshots of the instantaneous phases (left panel) and the effective frequencies (right panel) for the linear correlation when the intra-layer and inter-layer coupling strength are set to 0.7 and 0.5, respectively.

Fig 2a and 2b show that for the linear correlation, the system also induces a chimera-like state. For the homogeneous coupling and the linear correlation, the processes of phase locking and frequency synchronization of coupled oscillators are similar, that is, the central region is synchronized first, and then extends to both ends. It can be inferred that for the sub-linear correlation, the synchronization transition of the system should be gradual rather than abrupt.

In order to characterize the coherence degree of the coupled oscillators in each layer, two separate phase order parameters are considered [33],

$$\begin{array}{c} R^{u(l)}e^{i\psi^{u(l)}}=\frac{1}{N}\sum_{j=1}^{N}e^{i\phi_{j}^{u(l)}} \end{array}$$ (3)

where *R*^*u*(*l*)^*ϵ*[0, 1] is a measure of the coherence of coupled oscillators in the upper (lower) layer. The greater its value, the higher the coherence. The parameter *ψ*^*u*(*l*)^ is the average phase of coupled oscillators in the upper (lower) layer.

In the following numerical simulations, Eq 1 is integrated by the fourth-order Runge-Kutta method with time step 0.01. Excluding the randomness of initial phases, the network parameters of the upper and lower layers are exactly the same. In order to avoid repetition, the evolution law of order parameter of the lower layer is omitted.

**Fig 3:**
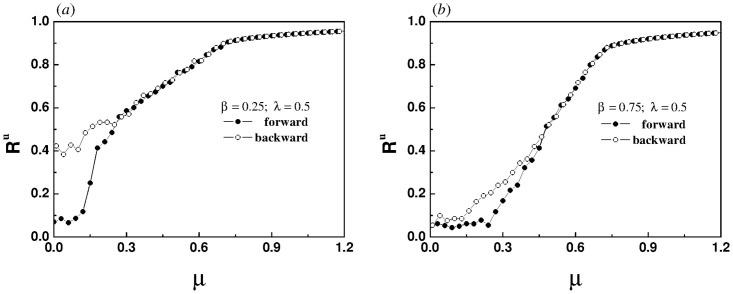
The evolution of order parameter of the upper layer with the increase of intra-layer coupling strength for different values of *β*, (a) *β* = 0.25, (b) *β* = 0.75. The inter-layer coupling strength λ is set to 0.5. Every data point in the two panels is the average of 2000 time steps after discarding the initial 2000 time steps.

Fig 3 shows that for the sub-linear correlation, a continuous phase transition does occur in the system, which confirms our above inference. However, due to the strong coupling between layers, the system cannot return to the original state after desynchronization. The smaller the value of *β*, the worse the desynchronization performance, as shown in Fig 3a.

### Emergence of explosive synchronization

**Fig 4:**
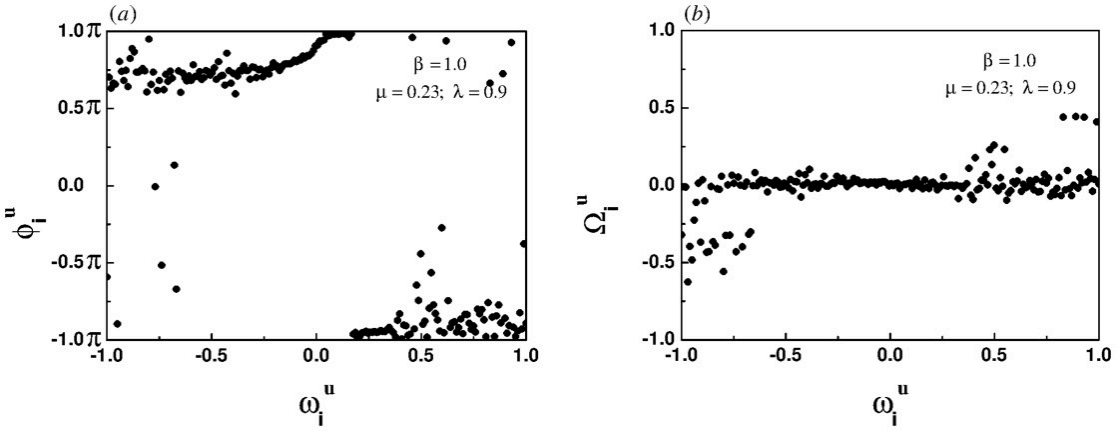
Snapshots of the instantaneous phases (left panel) and the effective frequencies (right panel) for the linear correlation when the intra-layer and inter-layer coupling strength are set to 0.23 and 0.9, respectively.

Fig 4a and 4b show the snapshots of instantaneous phases and effective frequencies of all oscillators in the upper layer under the case of linear correlation, respectively. As shown in Fig 4b, the effective frequencies of the oscillators located in the central region of the frequency spectrum are convergent, while the effective frequencies of the oscillators located at both ends of the spectrum are divergent, indicating the emergence of a chimera-like state.

**Fig 5:**
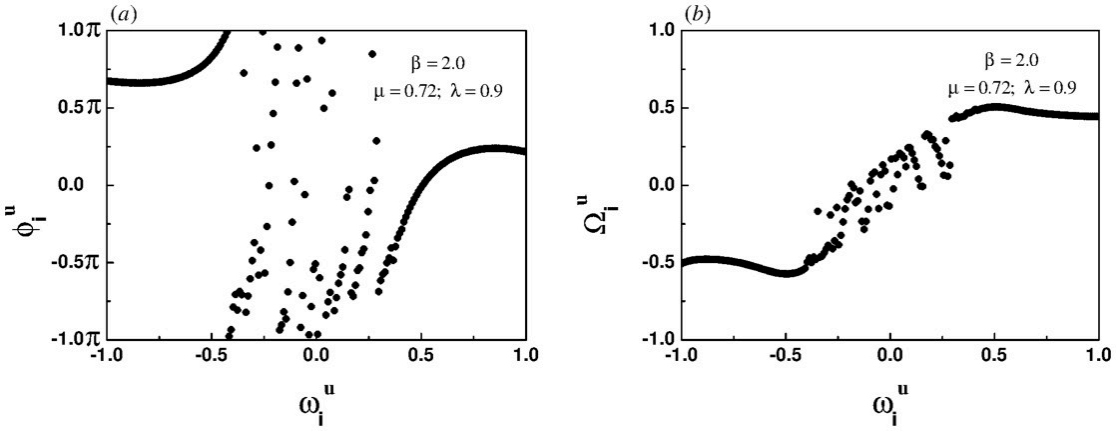
Snapshots of the instantaneous phases (left panel) and the effective frequencies (right panel) for the super-linear correlation when the intra-layer and inter-layer coupling strength are set to 0.72 and 0.9, respectively.

Fig 5a and 5b show that for the super-linear correlation, the system also induces a chimera-like state. However, the process of phase locking and frequency synchronization first occurs at both ends, and then gradually spreads to the middle, which is just opposite to Fig 4.

For the case of *β* = 1, the frequency synchronization of coupled oscillators starts in the middle of the spectrum, but for *β* = 2, the frequency synchronization first occurs at both ends of the spectrum. We speculate that if *β* takes a value between 1 and 2, the oscillators in the system will not be able to determine the location where synchronization occurs first. Therefore, the oscillators in the system reach a consensus, either out of synchronization or collective synchronization, which leads to the emergence of explosive synchronization.

**Fig 6:**
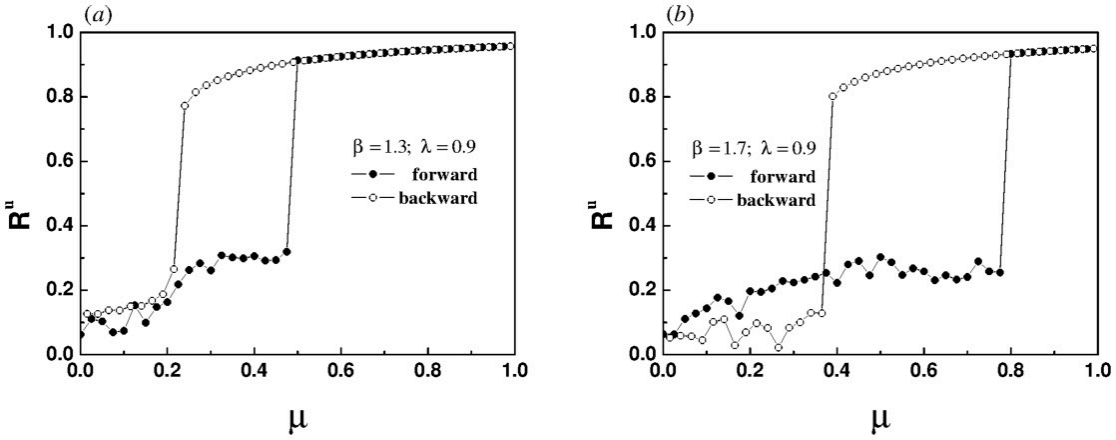
The evolution of order parameter of the upper layer with the increase of intra-layer coupling strength when the inter-layer coupling strength λ is set to 0.9. Both of them correspond to the super-linear correlations, (a) *β* = 1.3, (b) *β* = 1.7. Every data point in the two panels is the average of 2000 time steps after discarding the initial 2000 time steps.

To verify this speculation, both synchronization and desynchronization diagrams are plotted for two different values of *β*, as shown in Fig 6. For the super-linear correlation, the system does induce an explosive synchronization. However, due to the influence of inter-layer coupling, the system can not return to the original state after desynchronization.

For the super-linear correlation, why does the network induce an explosive synchronization? Our theoretical explanation is as follows. The frequencies of the oscillators who located in the central region of the spectrum of natural frequency are close to the frequency of ensemble equilibrium state, so they are most likely to evolve into condensation nuclei of network synchronization, that is, to achieve synchronization first. However, the oscillators at both ends of the spectrum enjoy greater relative inter-layer coupling strength. Here, the relative inter-layer coupling strength refers to the ratio of the absolute inter-layer coupling strength to the positive natural frequency of the frequency dipoles, i.e., $\lambda_{r}=\lambda |\omega_{i}^{u}{|}^{\beta }/|\omega_{i}^{u}|=\lambda |\omega_{i}^{u}{|}^{1-\beta }$. Due to the difference of advantages, each oscillator in the network has equal opportunity to evolve into the first synchronous condensation center. As long as their advantages are balanced, an explosive synchronization will be ignited.

**Fig 7:**
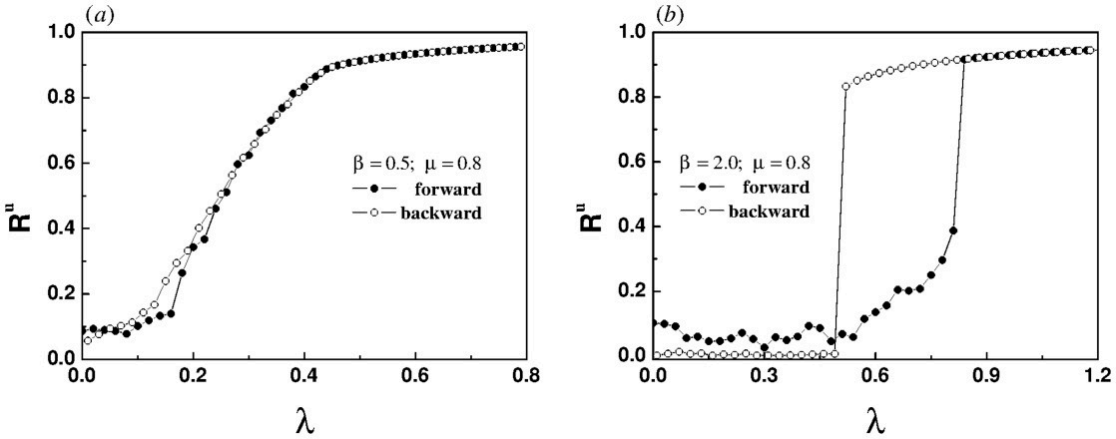
The evolution of order parameter of the upper layer with the increase of inter-layer coupling strength for different values of *β*, (a) *β* = 0.5, (b) *β* = 2.0. The intra-layer coupling strength *μ* is set to 0.8. Every data point in the two panels is the average of 2000 time steps after discarding the initial 2000 time steps.

Next, we keep the intra-layer coupling strength unchanged and study the synchronization transition characteristics of coupled oscillators by increasing the inter-layer coupling strength. The same law is found that the second-order phase transition occurs in the sub-linear coupled system, as shown in Fig 7a. It is easy to see that there is a hysteresis loop in the super-linear coupled system, indicating the emergence of explosive synchronization, as shown in Fig 7b. It is worth noting that the desynchronization effect is the most thorough in this system.

**Fig 8:**
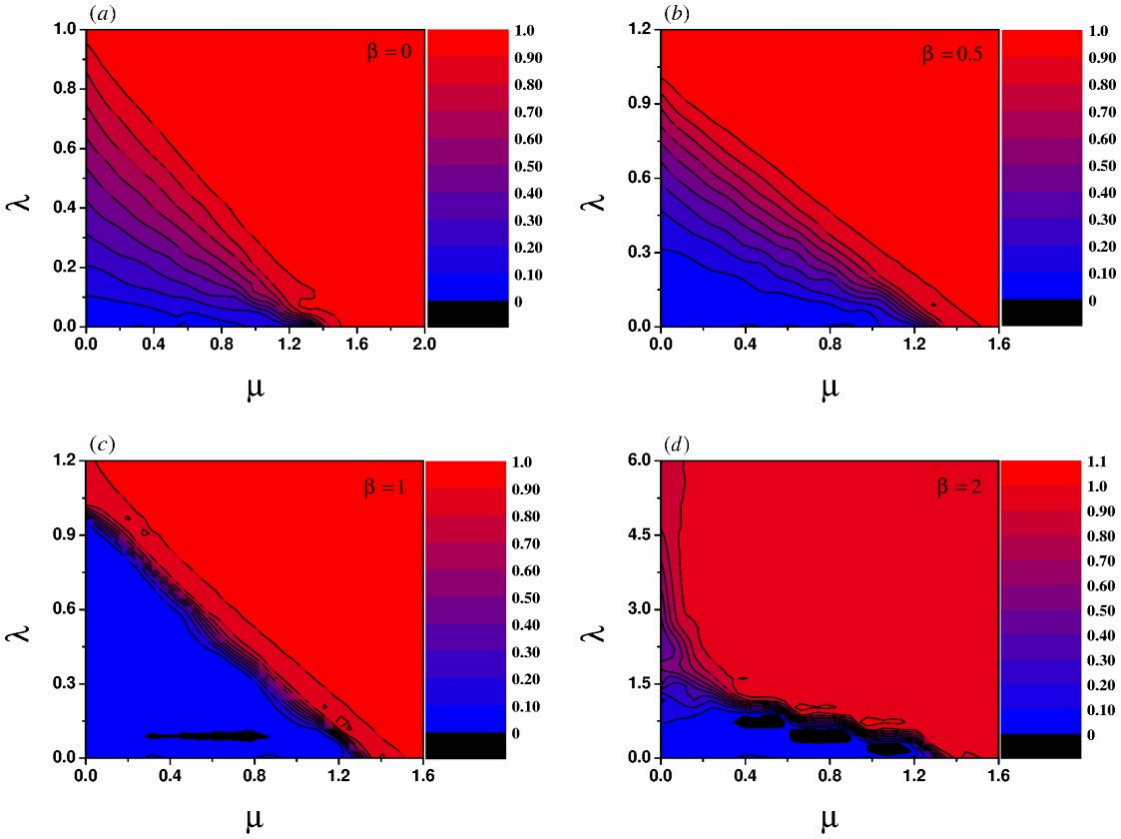
Order parameter of the upper layer is described by chromaticity in the λ − *μ* parameter plane. The chromaticity ranges from 0 (black) to 1 (red). The greater its value, the higher the degree of phase coherence. The values of *β* in each panel are (a) *β* = 0, (b) *β* = 0.5, (c) *β* = 1, (d) *β* = 2, respectively.

Now we systematically study the influence of power-law exponents on the synchronization transition of coupled oscillators. In Fig 8, the order parameter of the upper layer is described by chromaticity in the λ − *μ* parameter plane for different power-law exponents. It is generally realized that with the increase of the coupling strength within and between layers, the oscillators in the network tend to be synchronized. The simulation results are consistent with our intuition. For the homogeneous coupling, sub-linear correlation and linear correlation, the boundary lines between different degrees of synchronization is clear and recognizable, as shown in Fig 8a–8c. Nevertheless, for the super-linear correlation the boundary lines between different degrees of synchronization are intertwined and even distorted in certain areas as shown in Fig 8d, which may be the internal cause of the emergence of explosive synchronization.

**Fig 9:**
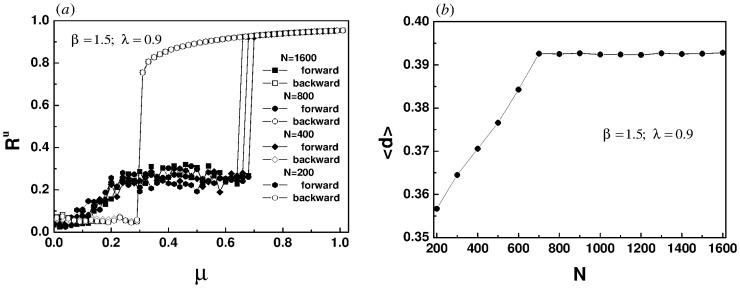
(a) Synchronization and desynchronization diagrams of upper layers with different sizes. (b) Dependence of hysteresis loop width on network size. Other parameters are set to *β* = 1.5 and λ = 0.9.

Let us now investigate the influence of system size on the hysteresis loop width. Fig 9a shows the forward and backward continuous diagrams of upper layers of different sizes. It is found that the width of the hysteresis loop increases with the size of the system until it reaches saturation. However, there is an interesting phenomenon in the process of backward transition. As shown in Fig 9a, the critical point of the backward continuation in the model is robust, which does not change with the increase of system size [14, 16]. For each size, there is a pair of corresponding forward and backward transition points *μ*_*f*_ and *μ*_*b*_, with *d* = *μ*_*f*_ − *μ*_*b*_. Fig 9b reports the corresponding dependence of < *d* > on *N* for twenty realizations. The simulation results show that when the size of the upper layer increases to *N* = 700 (the size of the lower layer changes synchronously) the width of the hysteresis loop tends to be saturated, which also indicates that the characteristics of explosive synchronization in the system have not changed. Fig 9 verifies that an increase in system size does not change the type of synchronization transition.

### Theoretical analysis

In this section, we give the critical values of intra-layer and inter-layer coupling strength in two limit cases from the perspective of theoretical analysis. Considering the case of λ = 0, the bilayer network is split into two incoherent globally connected networks. The critical value of intra-layer coupling strength is obtained [33],

$$\begin{array}{c} \mu_{c}=\frac{2}{\pi g(0)}=\frac{4}{\pi }\approx 1.27 \end{array}$$ (4)

where *g*(⋅) is a probability distribution function. The four panels in Fig 8 show that the position where the blue area disappears on the horizontal axis occurs at about *μ* = 1.3. Obviously, our theoretical analysis is consistent with the numerical simulation.

When the connection between the two layers is restored, the average coupling strength of each edge is given by,

$$\begin{array}{c} \bar{\lambda }=\frac{1}{N}\sum_{i=1}^{N}\lambda |\omega_{i}^{u(l)}{|}^{\beta }\approx \frac{1}{N}\frac{2\int_{0}^{1}\lambda \omega^{\beta }d\omega }{2/N}=\frac{\lambda }{1+\beta } \end{array}$$ (5)

Eq 5 shows when the values of λ and *μ* remain unchanged, the increased *β* weakens the synchronization ability of the network. When the value of *β* increases, it causes the intersections between the boundary lines with different degrees of synchronization and the longitudinal axis to move upward, as shown in Fig 8.

The left and right sides of Eq 3 are multiplied by a factor $e^{-i\phi_{i}^{u(l)}}$ and substituted into Eq 1. Note that the first *i* in the factor is an imaginary unit and the second *i* is the label of the oscillator. Eq 1 can be rewritten as,

$$\begin{array}{c} \frac{d\phi_{i}^{u}}{dt}=\omega_{i}^{u}+\mu R^{u}sin\left(\psi^{u}-\phi_{i}^{u}\right)+\lambda |\omega_{i}^{u}{|}^{\beta }sin\left(\phi_{i}^{l}-\phi_{i}^{u}\right),\\ \frac{d\phi_{i}^{l}}{dt}=\omega_{i}^{l}+\mu R^{l}sin\left(\psi^{l}-\phi_{i}^{l}\right)+\lambda |\omega_{i}^{l}{|}^{\beta }sin\left(\phi_{i}^{u}-\phi_{i}^{l}\right), \end{array}$$ (6)

We mainly discuss the influence of inter-layer coupling on synchronization transition of the network in the case of *μ* → 0. Except that the distribution of initial phases is slightly different due to randomness, the other parameters of the upper and lower layers are exactly the same. Therefore, these equations hold after the system reaches a stable synchronous state, i.e., $d\phi_{i}^{u}/dt=d\phi_{i}^{l}/dt$, *R*^*u*^ = *R*^*l*^ and *ψ*^*u*^ = *ψ*^*l*^. Ignoring the intra-coupling term, Eq 6 for a pair of frequency dipoles becomes,

$$\begin{array}{c} 0=\omega_{i}^{u}+\lambda |\omega_{i}^{u}{|}^{\beta }sin\left(\phi_{i}^{l}-\phi_{i}^{u}\right),\\ 0=\omega_{i}^{l}+\lambda |\omega_{i}^{l}{|}^{\beta }sin\left(\phi_{i}^{u}-\phi_{i}^{l}\right), \end{array}$$ (7)

Thus the relationship between phase difference and the inter-coupling strength can be rewritten as,

$$\begin{array}{c} \lambda =-|\omega_{i}^{u}{|}^{1-\beta }/sin\left(\phi_{i}^{l}-\phi_{i}^{u}\right)\;\omega_{i}^{u}>0,\\ \lambda =|\omega_{i}^{l}{|}^{1-\beta }/sin\left(\phi_{i}^{u}-\phi_{i}^{l}\right)\;\omega_{i}^{l}<0, \end{array}$$ (8)

In view of the boundedness of sinusoidal function, it is easy to obtain the critical value of λ,

$$\begin{array}{c} \lambda_{c}=|\omega_{i}^{u(l)}{|}^{1-\beta } \end{array}$$ (9)

**Fig 10:**
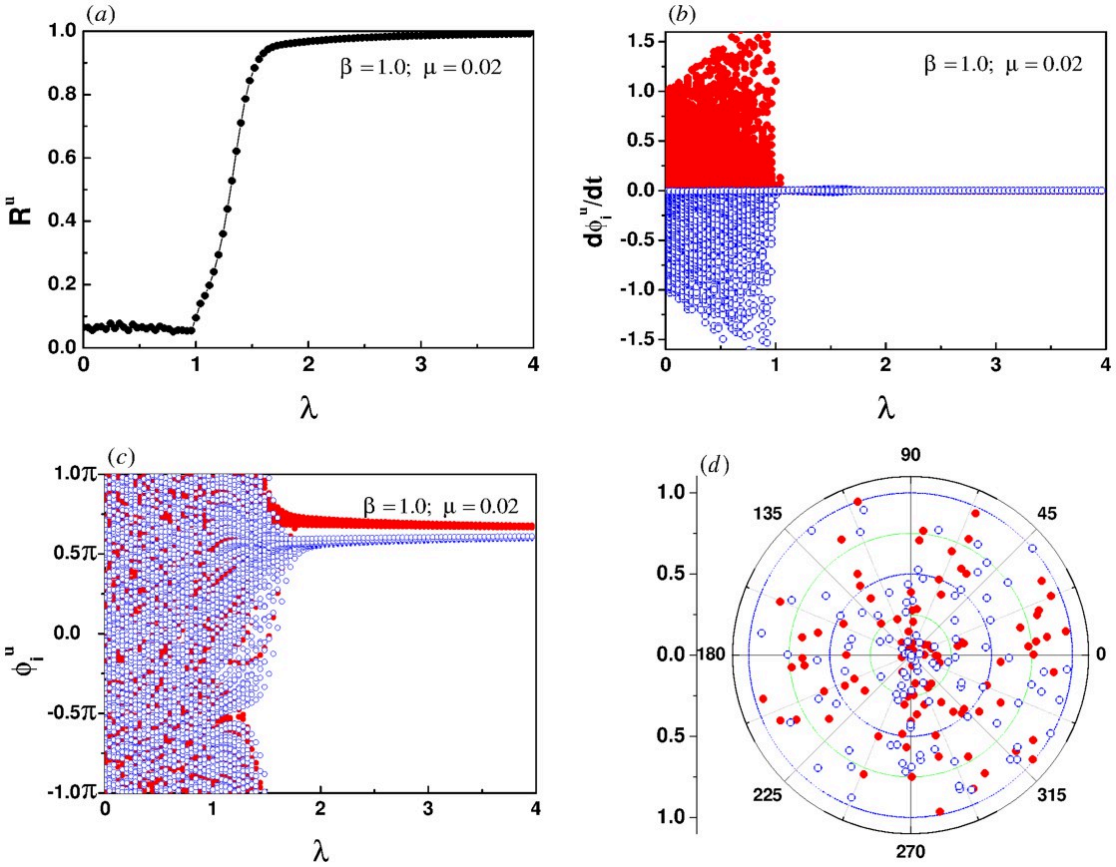
(a) The evolution of order parameter of the upper layer for the linear correlation. (b) and (c) show the evolutions of instantaneous frequencies and phases of the oscillators, respectively. (d) is the snapshot of instantaneous phases at λ = 1.0. The intra-layer coupling strength is set to 0.02. The red solid circles represent the oscillators with positive natural frequencies, while the oscillators with negative natural frequencies are described by blue hollow circles.

Fig 10a shows the dependence of *R*^*u*^ on λ in the case of *β* = 1. It is easy to see that the order parameter undergoes a continuous transition, where the incoherent state with *R*^*u*^ ≈ 0 is destabilized via a supercritical Hopf bifurcation at λ = 1.0 [34]. It is in perfect agreement with the theoretical result of Eq 9. The simulation results show that in this case the instantaneous frequencies of all oscillators have been synchronized as shown in Fig 10b, and the phase locking has just started, as shown in Fig 10c. Fig 10d exhibits the detailed characteristics of phase distribution of the oscillators. The oscillators are almost evenly scattered on the circular plane, the phases are unlocked, and the order parameter tends to zero in the macro view. The process of phase locking continues until the phases of all oscillators are locked when λ ≈ 1.8, as shown in Fig 10c.

## Discussion

**Fig 11:**
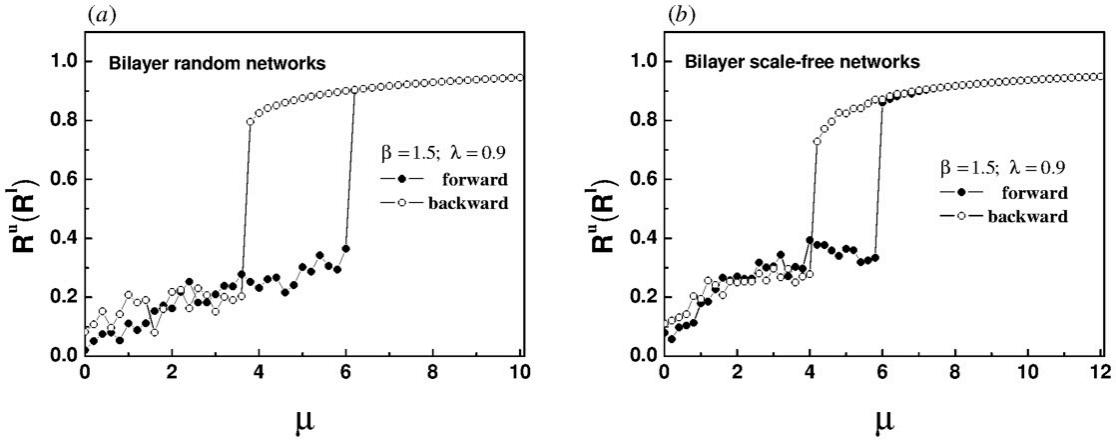
The panels show the evolution of order parameter of the upper layer with the increase of intra-layer coupling strength for different network topologies, (a) both the upper and lower layers are random networks, (b) both the upper and lower layers are scale-free networks. The inter-layer coupling strength λ is set to 0.9. The natural frequency of each oscillator in the upper layer is randomly selected in the interval [-1, 1], and the natural frequency of oscillators with the same label in the lower layer changes synchronously. Because the upper and lower networks belong to the same type, the evolution of order parameters is almost the same. In order to avoid repetition, the evolution law of order parameter of the lower layer is omitted.

**Fig 12:**
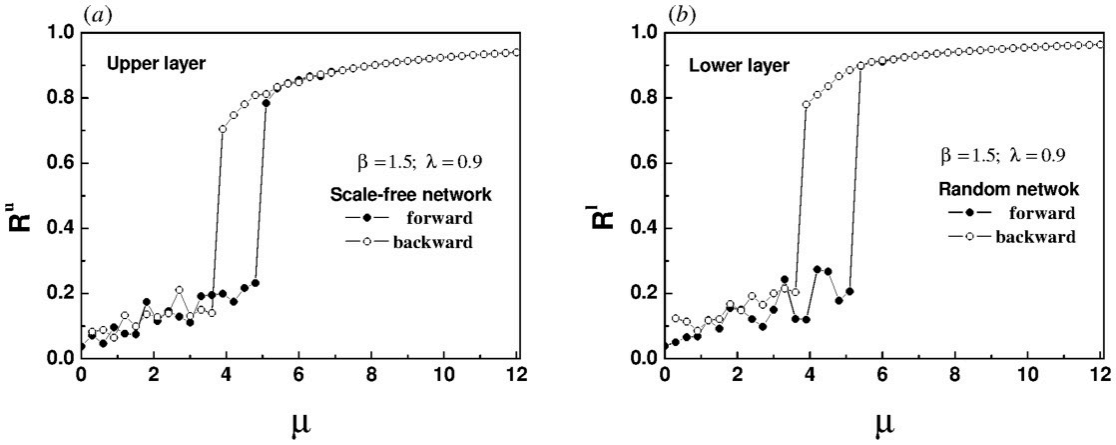
The panels show the evolution of order parameters of the upper (a) and lower (b) layers with the increase of intra-layer coupling strength for a bilayer network composed of a scale-free network in the upper layer and a random network in the lower layer. The inter-layer coupling strength λ is set to 0.9. The natural frequency of each oscillator in the upper layer is randomly selected in the interval [-1, 1], and the natural frequency of oscillators with the same label in the lower layer changes synchronously.

Furthermore, for the super-linear correlation we reconstructed the bilayer network, in which both the upper and lower layers of Fig 11a are random networks, both the upper and lower layers of Fig 11b are scale-free networks, the upper layer of Fig 12 is a scale-free network, and the lower layer is a random network. For all subsequent simulations, each layer of the network has 200 nodes and 2000 edges. The natural frequency of each oscillator in the upper layer is no longer forcibly specified, but is randomly selected within the interval [-1, 1]. The essence of a pair of frequency dipoles composed of oscillators with the same label in the upper and lower layers continues to be retained. The initial phase of each oscillator is randomly assigned within the range [−*π*, *π*]. Every data point in Figs 11 and 12 is the average of 2000 time steps, after discarding the initial 2000 time steps.

Obviously, hysteresis loops appear in the four panels of Figs 11 and 12, indicating that an explosive synchronization has emerged in both the upper and lower layers. Whether the upper and lower layers are homogeneous or heterogeneous, the super-linear correlation will induce an explosive synchronization.

**Fig 13:**
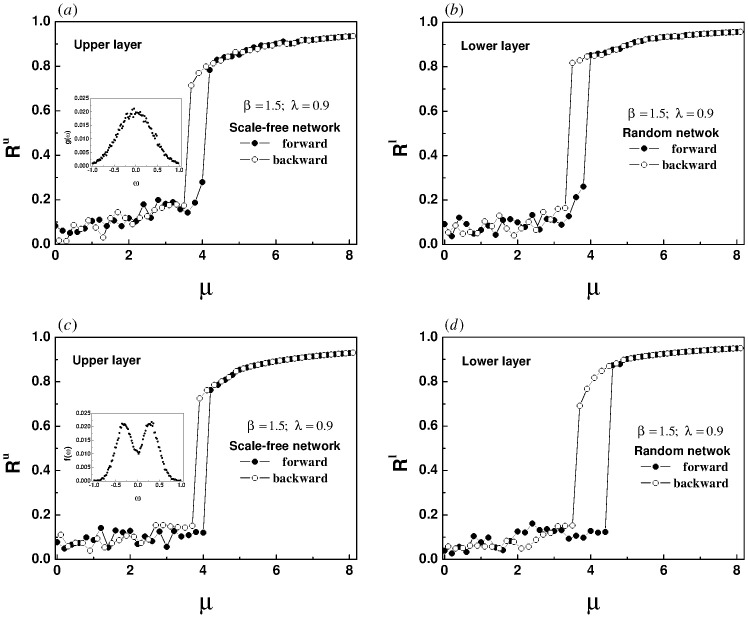
Synchronization and desynchronization transition diagrams of each layer at different natural frequency distributions. The topology of the network is exactly the same as Fig 12. We replace the random uniform distribution by two symmetric frequency distributions. The small inset in Fig 13a is a unimodal Gaussian distribution, in which the parameters are set to *ω*_0_ = 0 and *σ*^2^ = 0.16. The small inset in Fig 13c is a bimodal Gaussian distribution, in which the parameters are set to *A* = 20.3108, *B* = 0.1667, *ω*_0_ = 0 and *σ*^2^ = 0.0566.

Next, we use two different natural frequency distributions and maintain the same network topology as in Fig 12. Here two typical symmetric distributions, unimodal and bimodal Gaussian distributions, are considered. Fig 13 plots the dependence of *R*^*u*^(*R*^*l*^) and λ for two different natural frequency distributions. In Fig 13a and 13b, we replace the random uniform distribution by a unimodal Gaussian distribution. The probability density function $g\left(\omega \right)=\frac{1}{\sqrt{2\pi }\sigma }e^{\frac{-{\left(\omega -\omega_{0}\right)}^{2}}{2\sigma^{2}}}$ satisfies the symmetry condition, in which *ω*_0_ = 0 and *σ*^2^ = 0.16, as shown in the small inset of Fig 13a. The bimodal Gaussian distribution follows $f\left(\omega \right)=A\left(\omega^{2}+B^{2}\right)e^{\frac{-{\left(\omega -\omega_{0}\right)}^{2}}{2\sigma^{2}}}$, with *A* = 20.3108, *B* = 0.1667, *ω*_0_ = 0 and *σ*^2^ = 0.0566, as shown in the small inset of Fig 13c. Fig 13 reproduces the same scene as Fig 12. For these two different symmetrical frequency distributions, explosive synchronizations are also activated, independent of network topology. In addition, two kinds of asymmetric frequency distributions, power-law distribution and Rayleigh distribution are tested, which failed to trigger an explosive synchronization. The corresponding synchronization and desynchronization diagrams are not given in this paper. We conclude that only when the natural frequencies of the oscillators satisfy the symmetric distribution, the super-linear correlation can excite an explosive synchronization.

## Conclusion

In this paper, inspired by the idea of the electric dipoles and frequency weighting, the coupling-frequency correlations are introduced into a symmetric two-layer network. The effects of three typical correlations on synchronization transition of coupled oscillators are studied in detail.

For the sub-linear correlation, the oscillators in the center of the frequency spectrum have two superiorities in the synchronization transition. On the one hand, their natural frequencies are closer to the steady state of the ensemble, which makes them locked first. On the other hand, they obtain greater relative inter-layer coupling, in which the relative inter-layer coupling refers to the ratio of absolute inter-layer coupling to the natural frequency. The two superiorities contribute to the emergence of chimera-like states.

For the linear correlation, there is no difference in relative inter-layer coupling strength. Naturally, the oscillators whose natural frequencies are closer to the steady state of the ensemble tend to be synchronized first, resulting in chimera-like states.

For the super-linear correlation, the oscillators located at the center of the frequency spectrum have the location advantage, while the oscillators in the ends of frequency spectrum have the superiority of larger relative inter-layer coupling strength. When the two superiorities are well-matched in strength, an explosive synchronization occurs. Therefore, the network of frequency dipoles can be considered as one of the effective models to describe the complex system where chimera-like states and explosive synchronization are pervasive.

## Supporting information

S1 Data Minimal data set. This compressed file covers the data information of all pictures in the manuscript.
(ZIP)
